# Interoperability opportunities and challenges in linking mhealth applications and eRecord systems: Botswana as an exemplar

**DOI:** 10.1186/s12911-021-01606-7

**Published:** 2021-08-21

**Authors:** Kagiso Ndlovu, Richard E. Scott, Maurice Mars

**Affiliations:** 1 Department of TeleHealth, School of Nursing and Public Health, College of Health Sciences, University of KwaZulu-Natal, Durban, South Africa; 2 Department of Computer Science, University of Botswana, Gaborone, Botswana; 3 Department of Community Health Sciences, Cumming School of Medicine, University of Calgary, Calgary, AB Canada; 4 College of Nursing and Health Sciences, Flinders University, Adelaide, South Australia Australia

**Keywords:** eHealth, mHealth applications, eRecord systems, Interoperability, Challenges, Opportunities, Botswana

## Abstract

**Background:**

Significant investments have been made towards the implementation of mHealth applications and eRecord systems globally. However, fragmentation of these technologies remains a big challenge, often unresolved in developing countries. In particular, evidence shows little consideration for linking mHealth applications and eRecord systems. Botswana is a typical developing country in sub-Saharan Africa that has explored mHealth applications, but the solutions are not interoperable with existing eRecord systems. This paper describes Botswana’s eRecord systems interoperability landscape and provides guidance for linking mHealth applications to eRecord systems, both for Botswana and for developing countries using Botswana as an exemplar.

**Methods:**

A survey and interviews of health ICT workers and a review of the Botswana National eHealth Strategy were completed. Perceived interoperability benefits, opportunities and challenges were charted and analysed, and future guidance derived.

**Results:**

Survey and interview responses showed the need for interoperable mHealth applications and eRecord systems within the health sector of Botswana and within the context of the National eHealth Strategy. However, the current Strategy does not address linking mHealth applications to eRecord systems. Across Botswana’s health sectors, global interoperability standards and Application Programming Interfaces are widely used, with some level of interoperability within, but not between, public and private facilities. Further, a mix of open source and commercial eRecord systems utilising relational database systems and similar data formats are supported. Challenges for linking mHealth applications and eRecord systems in Botswana were identified and categorised into themes which led to development of guidance to enhance the National eHealth Strategy.

**Conclusion:**

Interoperability between mHealth applications and eRecord systems is needed and is feasible. Opportunities and challenges for linking mHealth applications to eRecord systems were identified, and future guidance stemming from this insight presented. Findings will aid Botswana, and other developing countries, in resolving the pervasive disconnect between mHealth applications and eRecord systems.

## Background

eHealth is defined by the World Health Organization (WHO) as “the use of Information and Communications Technologies (ICT) for health” [1] and comprises four application areas (health informatics, telehealth, e-learning, and e-commerce [2]). mHealth, a component of telehealth, is “the use of mobile communications for health information and services” [3], and mHealth applications (ways in which all modes of mHealth hardware, software, infrastructure, and connectivity can be used for specific purposes, not merely ‘apps’) are used extensively worldwide to address many health and healthcare needs [4].

Like mHealth, the use and implementation of electronic record (eRecord) systems such as electronic health records (EHR), electronic medical records (EMR) or personal health records (PHR) is growing rapidly. By 2017, 84% of hospitals, 93% of clinics and 96% of general practitioners in the European Union (EU) used an electronic patient record system [5], and in the United States 99% of hospitals [6] and 86% of office-based physicians had adopted an EHR [7]. The situation in the developing world is somewhat different. Fewer than 15% of low income countries have been reported to have a national EHR [8], although developing countries have implemented various programme or disease specific eRecord systems to augment current paper-based methods [9, 10].

Similarly, differences exist in regard to mHealth. Developed countries predominantly use smartphones and have implemented formal mobile solutions, whereas in developing countries basic phones and feature phones still predominate and, given the lack of or limited regulations, mHealth is used more informally [11]. Thus, whilst sub-Saharan Africa (SSA) has been a focal point for many formal mHealth projects, informal use appears common [11, 12]. Such trends are likely to be spurred by recent global awareness and application of eHealth, including mHealth, due to the COVID-19 pandemic [13].

The benefits of eRecord systems are well documented, particularly the prevention of duplicate tests and improved quality of care related to accessing patient information between different sites as and when required [14]. The primary benefit of mHealth is mobility and the potential to use this to advantage in extending geographic access to healthcare in any location where there is connectivity. Linking of mHealth applications and eRecord systems should result in seamless data exchange within a healthcare community with subsequent benefit [15, 16].

Despite widespread adoption, interoperability of mHealth applications and eRecord systems remains a global challenge [14]. Interoperability is broad and has numerous definitions. The Healthcare Information and Management Systems Society’s (HIMSS) definition is the most encompassing, “the ability of different information systems, devices and applications (‘systems’) to access, exchange, integrate and cooperatively use data in a coordinated manner, within and across organizational, regional and national boundaries, to provide timely and seamless portability of information and optimize the health of individuals and populations globally” [17]. To this could be added “… without special effort on the part of the user”, from the definition of the Office of the National Coordinator for Health Information Technology [18].

Interoperability utilises standards, interfaces and protocols to connect systems using appropriate techniques, methodologies and all associated issues such as legislation, agreements, governance, workflows and privacy concerns. It can be achieved at various ‘levels’ (technical, syntactic, semantic, organisational and legal) [19, 20]. It can also be assessed in other ways. One approach is to use the FAIR guidelines to assess the capacity of computational systems to Find, Access, Interoperate, and Reuse (FAIR) data with minimal or no human intervention [21].

The implementation and adoption of eRecord systems and mHealth applications in SSA has been slow and the systems that are in place have usually been developed by donors for a single problem, such as HIV/AIDS, tuberculosis, or malaria. This has resulted in silos of eRecord systems often containing similar data on the same patients, and extensive ‘donor-driven pilotitis’ [22]. In 2010, Uganda had over 50 concurrent mHealth and eHealth projects (and almost as many donors) and at least a dozen eRecord systems, stimulating the Government to issue on 17th January 2012, a moratorium. This directed that “all eHealth projects/initiatives be put to halt until” specified national and institutional infrastructure was established [22]. A similar problem was highlighted in Botswana where, in 2013, the Ministry of Health and Wellness (MOHW) reduced 37 distinct eRecord systems to a more manageable nine [23]. Botswana has identified mHealth as a means of improving healthcare provision and delivery [9]. Past localised efforts have been made to use mHealth in four clinical areas [24], but none were linked to any eRecord system(s).

Provision of healthcare in Botswana is decentralised across an extensive network of health facilities, distributed throughout 27 health districts, that includes national referral hospitals, district hospitals, primary hospitals, clinics, health posts, and ‘mobile stops’ [23]. Diverse eRecord systems from different vendors and donors exist across public and private health sectors, and are not interoperable resulting in fragmented care delivery, duplication of effort and unnecessary healthcare expenditure.

Whilst Botswana’s recent eHealth Strategy addresses the development of an interoperability platform, it provides no guidance regarding interoperability of mHealth applications and eRecord systems. This is consistent with a recent systematic review of mHealth interventions in developing countries which concluded that “most of the mobile health interventions are not ready for interoperability and to be integrated into the existing health information systems” [25]. In addition, despite the proliferation of both mHealth applications and eRecord systems, the need for their bi-directional interoperability has not been fully appreciated or addressed in Botswana [26]. This shortcoming must be addressed.

In this paper, Botswana is used as an exemplar for developing world countries with a similar interoperability setting (particularly in SSA), and an approach to identifying and resolving mHealth and eRecord systems interoperability issues is described. The aim of this study was to engage with local eHealth experts about interoperability and linking mHealth applications to eRecord systems and to conduct a review of the Botswana National eHealth Strategy. The goal was to inform and expand interoperability approaches available to developing countries and to facilitate enhancement of Botswana’s National eHealth Strategy in particular.

## Methods

A survey and key informant interviews (KII) of health information and communication technology (HICT) personnel were conducted, and the Botswana eHealth Strategy was reviewed to identify considerations relevant to interoperability of mHealth and eRecord systems. The aspects of the eHealth Strategy related to interoperability were summarised and charted to align aspects of the survey and KII findings with the proposed development of an interoperability platform for the country. Collectively these provide an informed context for the linking of mHealth applications to eRecord systems.

The authors developed a questionnaire addressing: the eRecord systems in place at respondents’ institutions; the standards upon which the systems are based; the data formats and application programming interfaces (APIs) supported by the systems; the interoperability of their systems; their understanding of interoperability and associated challenges; how their data are organised and identified; alignment with the FAIR guidelines; and government support for their mHealth applications and eRecord systems. The survey had 23 closed-ended questions (2 dichotomous; 7 multiple choice; 14 Likert scale) and 19 open-ended questions. A 3-point Likert scale was used for the 14 closed-ended questions related to FAIR guidelines (ordinal scale: Yes = 1, Unsure = 2 and No = 3). The survey questionnaire was first reviewed by a medical faculty member and a medical librarian and refined to avoid noted ambiguities. It was then pre-tested by two HICT personnel before being enhanced through improved branching logic. The refined survey was administered online using the Research Electronic Data Capture (REDCap) forms from 24 September, 2019 to 23 March, 2020.

Eighteen HICT personnel (IT officers or technicians, IT managers, system analysts, and health informaticians) were purposively sampled from seven public hospitals in Gaborone (3), Francistown (3) and Maun (1); three private hospitals in Gaborone (3); and six clinics, one public and one private in each of the three districts surveyed. These locations were selected because eRecord systems are used in these districts. Each of the clinics had only one person responsible for IT who participated, while at hospitals senior IT personnel were recruited. In addition, the three most senior members of the MOHW (IT personnel and health informatics personnel) also completed the survey.

Eight key informant interviewees were also purposively selected from the survey respondents (four IT officers, two systems analysts, one health informatician, and one health informatics director) from across the healthcare facilities. The semi-structured interviews sought further clarity on key issues identified in the surveys and covered questions on: eRecord system readiness to interoperate with mHealth applications, the level of interoperability to be achieved, perceived benefits and barriers to mHealth applications and eRecord systems interoperability, interoperability standards, APIs, and security considerations. All interviews were recorded and transcribed verbatim, transcripts entered into NVivo and analysed for themes. Final themes were agreed by consensus of all authors. Descriptive statistics were used and calculations of mean, standard deviation and median values reported.

## Results

### Survey findings

Fifteen of the 18 HICT personnel responded (83%). The three non-respondents were from two public hospitals (Francistown and Maun regions) and one private hospital in the Gaborone region. Respondents were from the MOHW (3), public hospitals (4), private hospitals (2), public clinics (3) and private clinics (3). Their roles included health informatician (4), information technology specialist (6), and systems analyst (5).

### Available mHealth and eRecord systems

All surveyed health facilities had either one or more eRecord systems in place, and only one public hospital supported an mHealth application. All public hospitals accessed the Integrated Patient Management System (IPMS), District Health Information System (DHIS) and the Open-Medical Record System (OpenMRS), and public clinics accessed the IPMS and a stand-alone Patient Information Management System (PIMS). One public hospital also accessed the mHealth store and forward telemedicine system (Kgonafalo). Private hospitals hosted different eRecord systems and used them to support both clinical services and medical billing processes. The systems in use are shown in Table 1.

**Table 1:** mHealth and eRecord systems in use at surveyed health facilities

eRecord systems/mHealth applications	Description	DBMS	Public Hosp. (n = 7)	Private Hosp. (n = 2)	Public clinics (n = 3)	Private clinics (n = 3)	Total
Integrated Patient Management System (IPMS)	An integrated and centralised patient medical record system with 12 interoperable modules. Currently at all public hospitals, and some clinics	Microsoft SQL (Relational)	7	–	3	–	10
Patient Management Information Systems (PIMS)	A standalone system for management of HIV patient’s medical records and used in clinics and health posts, which do not have IPMS	Microsoft SQL (Relational)	–	–	3	–	3
R-Care	Health information system which supports clinical, financial and procurement modules	Procedural Languages PL/SQL (Relational)	–	1^a^	–	–	1
Sukraa Hospital Information Management System	Sukraa health information system supporting all clinical data management and linked with the SAP suite for supporting the billing module	Microsoft SQL (Relational)	–	1^a^	–	–	1
Laboratory Information Management System (Senaite)	An open source system customised for specific laboratory workflows (complete circle of laboratory specimen sample)	Zope Object Oriented database (Non-relational)	–	–	–	1	1
TriMed systems	Patient administration services from registration to discharge from the hospital, including billing and medications	Microsoft SQL (Relational)	–	1^b^	–	–	1
District Health Information System (DHIS2)	An open source Web based system used for capturing aggregated data on patient diagnosis at each of the health districts	Postgre-SQL (Relational)	7	–	–	–	7
OpenMRS	An open source system for capturing/storage of TB patient’s data	MySQL (Relational)	7	–	–	–	7
Mobile Telemedicine (Kgonafalo)	mHealth application to communicate patient data and images between facilities and specialists at referral hospitals. Services included: oral medicine, radiological services, dermatology and cervical cancer screening	MySQL (Relational)	1	–	–	–	1
Arcus Air (web based)	Used within medical wards (EMR) for medical record keeping and related clinical services	MongoDB (Non-relational)	–	1^b^	–	–	1
Optima	Used for patient registration, billing and report generation	Microsoft SQL (Relational)	–	–	–	3	3

DBMS—database management system

SQL – Structured Query Language

^a, b^—refer to two Private hospitals that both used two different systems

The MOHW site was not considered a healthcare facility. One MOHW respondent did not have access to an eRecord system. The other two used an open-source cancer registry, and a central medical stores (CMS) warehouse management system for inventory management of medicines and medicinal products, both of which make use of Structured Query Language (SQL) databases.

### mHealth and eRecord system characteristics

The characteristics of the eRecord systems currently in use (classification, interoperability standards, data formats, and APIs) are shown in Table 2.

**Table 2:** Reported characteristics of the mHealth application and eRecord systems: classification, standards, data formats, and APIs

Facility	eRecord Systems Classification	Standards	Data Formats	APIs
MOHW	Both open source and commercial	HL7	PDF, HTML, PlainText, JPEG, BMP, GIF, XML, CSV	GxAlert
Public Hospital	Both open source and commercial	DICOM, HL7, ISO/IEEE 11073	PDF, HTML, PlainText, JPEG, PNG, CSV	IPMS API (RESTful APIs)
Private Hospital	Both open source and Commercial	HL7 ISO/IEEE 11073 DICOM	PDF, HTML, PlainText, JPEG, PNG, XML, CSV	RESTful APIs
Public Clinic	Both open source and commercial	HL7	PDF, HTML, PlainText, JPEG, PNG, GIF, XML, CSV	RESTful APIs
Private Clinic	Commercial	.NET HL7 ISO/IEEE 11073	PDF, HTML, XML, CSV	HL7/XML based API

Participants reported that past implementation of mHealth applications and eRecord systems in the public sector were based on the expectations of the then draft National eHealth Strategy, while within the private sector eRecord systems are implemented based on in-house standard operating procedures (SOPs) [27], and some followed the Southern African Development Community Accreditation Services (SADCAS) standards [28].

Four HICT personnel reported eRecord systems at their facilities to be technically interoperable; two at private hospitals, and one each at a public and private clinic. Three reported their systems non-interoperable, one each from two public clinics and a private clinic. At two public hospitals, not all eRecord systems were interoperable with other eRecord systems at the facility. HICTs at two public hospitals and one private clinic did not know if their eRecord systems were interoperable.

The Trimed and Arcus Air eRecord systems as well as the Sukraa and the Services Applications and Products (SAP) system, used at two private hospitals respectively, were reported to be HL7 compliant and envisaged to be interoperable. Of note was that the mHealth application (Kgonafalo), although not linked to any EMR, was also HL7 compliant and the SAP system supported an HL7 Integration Adapter Healthcare module, which enables translation of HL7 XML to native HL7 v2 pipe format structure.

The IPMS was reported to support twelve interoperable modules of which only the Admissions, Laboratory and Microbiology modules had been implemented. The Optima eRecord system adopted at private clinics was reported to be a cloud-based solution and interoperable with other eRecord systems such as MatrixCare, PointClickCare and HealthMEDX. A Laboratory Information Management System (Senaite) at a private clinic in the Gaborone region had a customisable API allowing for interoperability with other eRecord systems.

### Alignment to FAIR guidelines

FAIR guiding principles were used to assess the capacity of eRecord systems to ***F***ind, ***A***ccess, ***I***nteroperate, and ***R***euse data with no or minimal human intervention. Of the responses to the FAIR guidelines, the strongest positive response was to Findable. For 6 of the remaining 10 variables, half of respondents were unsure of aspects related to Accessibility, Interoperability and Reusability. In response to the questions on the FAIR guiding principles the respondents showed limited understanding of the features of their eRecord systems. The mean percentage of unsure responses ranging from 21.4 to 48.2% for the four criteria. The only overall positive response was for ‘Findable’ which had a mean score of just 53.6% with three of the four questions scoring 50% or more. Overall, the private sector responses to the FAIR guideline questions were positive, and came from the health informatician and information technology officers at 2 private hospitals which supported HL7 compliant eRecord systems (R-Care, Sukraa Hospital Information Management System, TriMed systems and Arcus Air (web based)). Two public hospitals also responded positively to FAIR guidelines and both supported the IPMS, DHIS2 and OpenMRS, all of which are interoperable via the HL7 standard. One public hospital also supported the mobile telemedicine application (Kgonafalo). The HICT personnel’s responses in regard to alignment of mHealth applications and eRecord systems data to FAIR Guidelines are summarised in Table 3.

**Table 3:** Responses to the FAIR Guidelines expressed as the number and percentage of responses, their mean, and the median score for each question, (n = 14)

	Sub-category	Yes n (%)	Unsure n (%)	No n (%)	Median
Findable	Are data assigned a globally unique and persistent identifier?	9 (64.3)	2 (14.3)	3 (21.4)	1
	Are data described with rich metadata?	7 (50.0)	3 (21.4)	4 (28.6)	2
	Do metadata clearly and explicitly include the identifier of the data it describes?	6 (42.8)	5 (35.7)	3 (21.4)	2
	Are data registered or indexed in a searchable resource?	8 (57.1)	2 (14.3)	4 (28.6)	1
	MEAN (%)	53.6	21.4	25.0	
Accessible	Are data retrievable by their identifier using a standardised communications protocol?	7 (50.0)	3 (21.4)	4 (28.6)	2
	Is the protocol open, free, and universally implementable?	2 (14.3)	7 (50.0)	5 (35.7)	2
	Does the system allow for an authentication and authorisation procedure, where necessary?	9 (64.3)	2 (14.3)	3 (21.4)	1
	Meta (data) are accessible, even when the data are no longer available?	3 (21.4)	7 (50.0)	4 (28.6)	2
	Mean (%)	35.7	33.9	28.6	
Interoperable	Data use a formal, accessible, shared, and broadly applicable language for knowledge representation?	7 (50.0)	3 (21.4)	4 (28.6)	2
	Data include qualified references to other (meta) data?	3 (21.4)	7 (50.0)	4 (28.6)	2
	Mean (%)	35.7	35.7	28.6	
Reusable	Meta(data) are richly described with a plurality of accurate and relevant attributes?	6 (42.8)	6 (42.8)	2 (14.3)	2
	(Meta)data are released with a clear and accessible data usage license?	3 (21.4)	7 (50.0)	4 (28.6)	2
	(Meta)data are associated with detailed provenance?	3 (21.4)	7 (50.0)	4 (28.6)	2
	(Meta)data meet domain-relevant community standards?	3 (21.4)	7 (50.0)	4 (28.6)	2
	Mean (%)	26.8	48.2	25.0	

### Perceived benefits of eRecord system interoperability

In response to open-ended questions, HICT personnel described their perception of the benefits of interoperable eRecord systems. These were: (1) easy flow and sharing of daily patient information and files among healthcare workers (HCWs), (2) automated data capture and an efficient data reporting interface across electronic health records (EHR), (3) potential to provide better healthcare for patients, (4) cost-effective delivery of healthcare services, (5) safer and patient centred healthcare, (6) retaining existing eRecord systems without losing legacy data, (7) minimised de-fragmentation of data, (8) improved continuity of healthcare services, (9) standardised data across all platforms, and (10) availability of patients' history whenever required by HCWs.

### eRecord systems interoperability challenges

Survey respondents reported multiple challenges to interoperability: lack of eRecord system legislation and governance, weak ICT infrastructure, limited maintenance budgets, lack of health human resource capacity to utilise eRecord systems, non-uniform unique patient identifiers (UPI), and non-enforcement of interoperability standards. Internet connectivity was reported as slow and irregular at the district level, with some facilities not having connectivity or electricity. The software upgrade policy was ineffective and required on-site installations, resulting in facilities running different versions of software. Budgets for Internet access or maintenance of eHealth devices were lacking. Due to inability to access the Government Data Network (GDN) at the District level, it was not possible to use the central data warehouse, with data being stored locally for later capture and transport to the MOHW computers. The lack of a national UPI resulted in patients having duplicate electronic and paper records.

### Governance and interoperability

A common interview finding was the importance of political and leadership support if interoperability of mHealth applications and eRecord systems is to be realised. Governance and regulation of eRecord systems were other reported challenges.

*“Political changes, unclear ownership of eHealth, inadequate policies and SOPs affects eRecord system implementation.”* (Health informatician)

*“Inadequate legal capacity, for contract management to protecting interests of providers and purchasers of systems, are challenges in Botswana.”* (System analyst)

*“Governance and legislative structures advance stakeholder coordination, collaboration and compliance with policies such as the Botswana Data Protection Act and related interoperability standards.”* (Health informatics director)

The lack of a single life-long UPI was raised by one participant:

*“The current identifiers (Omang and passport numbers) are not unique since several people could be assigned an Omang number and the passport number changes each time one renews their passport document.”* (IT officer)

A key finding from the interviews was greater awareness of the National eHealth Strategy within the public sector, including its pillar on interoperability, as well as plans to work with key stakeholders to implement the eHealth strategy between 2020 and 2024. In addition respondents had similar understandings of eRecord systems’ interoperability.

Of note from KII was that Botswana has made substantial investments in health ICTs within the public and private sectors, and despite lack of access at the District level, the majority of public sector HICTs were able to access a central data warehouse through the GDN. Reported opportunities for mHealth applications and eRecord systems and interoperability included the existence of; (1) semi-automated data generation and reporting processes, (2) a secured GDN network infrastructure, (3) robust mobile telecommunication infrastructure coupled with high use of mobile devices, (4) a national health data warehouse, and (5) common interoperability standards and APIs between existing eRecord systems and the mHealth application. The private sector was considered to be more advanced in its eRecord system implementation and use.

### Botswana’s national eHealth strategy review

The Botswana eHealth strategy, formally adopted in March 2020 [23], aligns with the World Health Assembly Resolution (WHA71.7) on Digital Health adopted in May 2018 [29] and key national policies including the Data Protection Act [30], the Botswana National Health Monitoring and Evaluation Plan [31], National Health Policy [32] and the Integrated Health Service Plan (IHSP) [33]. The strategy is built on seven pillars, one of which is ‘Standards and Interoperability’. The need for the development of an overall interoperability framework is seen as a priority, with an Open Health Information Exchange and an Open Health Information Mediator envisaged as facilitating the sharing of information between multiple individual systems. The strategy notes that, as a principle, international standards should be followed and open source solutions used where appropriate. The need for staff capacity development and improved infrastructure is also identified.

Key priority needs identified in the Strategy include *“Strengthening MOHW capacity to generate, disseminate, access, secure, store and use health information for evidence based planning at all levels and policy direction”* (subsection 1.3.2) and *“Establishing institutional/organizational arrangement that will harmonise and link all the data management units with the aim of reducing the duplication and wastage of data, and of mamixing its effective use through prompt reporting and feedback”* (subsection 1.3.2)*.* The Strategy does not use any of the terms ‘mHealth’, ‘personal health record’, ‘eRecord nor e-Record’. It is however stated that *“All public hospitals have an Electronic Medical Record (EMR) that is real time, …”.* Furthermore, the need to “*Establish a home-grown EHR for Botswana*” as a strategic intervention within a National eHealth Platform by 2023 was noted. The ‘Situational Analysis’ section recognised, although only indirectly, mHealth by noting a requirement for *“data available where and when it is needed”* and *“Data available on mobile devices”* (subsection 2.2.3). In addition the potential value of emerging technologies, including utilising mobile devices and IoT, is noted (subsection 2.2.3) as is the value of “*Sensors to populate digital devices with data*” (subsection 2.2.3). Importantly, the Strategy does not explicitly note the need to make mHealth applications interoperable with eRecord systems.

Alluding to interoperability issues, the ‘Standards and interoperability’ pillar seeks to strengthen health information availability and sharing by “*Establishing an interoperability architecture*” with specific strategic interventions of *“Establish[ing] a standards and interoperability framework”, “Design[ing] the interoperability platform”* and *“Implement[ing] the interoperability platform”* with key stakeholder involvement (subsection 3.5.4, Table 5 of the National eHealth Strategy [23])*.* Interoperability and the need to implement an overarching interoperability framework for Botswana is noted as a priority with “*the need for more interoperability and consolidation of existing information systems”* being described (subsection 2.2.3). The absence of an overarching health systems architecture was recognised as was the availability of “*interoperability architecture tools such as the Open Health Information Exchange (OpenHIE) and the Open Health Information Mediator (OpenHIM)*” (subsection 2.2.3). It was reported that *“The mediation layer will manage subsequent sharing of information between multiple individual systems.”* Infrastructure (workstation, Internet connectivity and server hosting) and human resource development needed to maintain and support the infrastructure were reported critical aspects for eHealth.

Activities contributing to infrastructure for completion by 2023, are to *“Connect all health facilities in the country with minimum bandwidth”, “Establish registries and national data dictionaries” and “Establishing Master Patient Index (MPI) for use by patients interfacing with the health system at all levels countrywide”.* Open source software endorsed by WHO and the need for a centralised national data repository (data-warehouse) were recognised. Consequently, performance, user friendly interfaces, human resource capacity building and availability of systems were emphasised. It was reported that *“Health data standards are critical to attain interoperability”*. Consequently, the Strategy will adapt existing WHO endorsed standards to the Botswana context (subsection 3.5.4) and enforce compliance across the health sector (subsection 3.5.4).

## Discussion

The study shows that even after a marked reduction in the number of eRecord systems in Botswana different databases are still used and most are still not interoperable within or between facilities or sectors, despite using widely accepted interoperability standards, data formats and APIs (Table 2), supporting the need for interoperability. Indeed data gathered from earlier mHealth telemedicine initiatives was not captured in an eRecord system, and is therefore no longer available; another demonstration of the impact of and need for interoperability. Furthermore, none are interoperable with the only mHealth application (Kgonafalo) supported by the MOHW. Respondents in the public sector were more aware of the National eHealth Strategy and the proposed interoperability platform than their counterparts in the private sector, whilst the opposite was true for understanding of interoperability concepts. However, the national eHealth Strategy does not identify the need to render mHealth applications and eRecords systems interoperable. It also emerged that the infrastructure of eRecord systems varied between the public and private health sectors, which do not share data and use different patient identifiers. Moreover, public facilities commonly use eRecord systems for patient record management while the private sector also used them to support hospital billing and store routine clinical data.

Challenges and considerations identified from the study for achieving interoperable mHealth applications and eRecord systems were categorised according to four themes: (1) eHealth legislation and governance, (2) eHealth software and infrastructure, (3) Data standards, security and UPI, and (4) Capacity building.

### eHealth legislation and governance

Interviewees reported political leadership and buy-in as important to drive linking of mHealth applications to eRecord systems. This aligns with previous studies which found that strong political and administrative buy-in could lead to the development of a robust health information exchange (HIE) and efficient governance structures that could influence cultural norms as well as stakeholders’ prioritisation of data and health information systems [34–37]. Corruption and unpredictable changes in policy have prevented successful implementation of eHealth [34]. The need for a Governance framework was also described in Botswana’s National eHealth Strategy.

Interviewees perceived the need for legislation to ensure stakeholder coordination, collaboration, and compliance with standards for eRecord systems and mHealth applications. Combined with coordinated compliance with the Botswana-specific Data Protection Act [30] (“the DPA”) such engagement could improve the necessary safeguards towards the right to privacy of individuals and the collection and transfer of their personal data from mHealth applications to and between eRecord systems in Botswana and across-borders. This finding is consistent with other studies which identified the lack of legislation for data confidentiality and ethics as key contributors to patient privacy and security concerns [34, 38–41].

### eHealth software and hardware infrastructure

Vendor lock-in to proprietary solutions causes difficulty when upgrading software [35, 38, 42]. Interviewees noted that different software versions exist across facilities, and their upgrade is time-consuming. In addition, software not developed in native languages [34] leads to language barriers and is an issue, as is software that is not designed with user preference and ease of use in mind [38, 41]. The Botswana eHealth Strategy promotes use of universally available software, services and content (global goods applications), endorsed by the WHO and designed to be interoperable [43]. Moreover, usability, availability, performance and user friendly interfaces of mHealth applications and eRecord systems are prioritised within the eHealth Strategy [23].

The GDN infrastructure was not accessible at all public facilities or to the private sector, although the Government has identified the need for the GDN to be updated and segmented, and consideration to be given to private sector access for data reporting. The lack of maintenance budgets to repair damaged hardware, lack of electricity in some facilities, frequent Internet downtimes affecting data capture and contributing to slow performance of eRecord systems were also reported, similar to previous studies in developing countries [34, 39, 41]. To support specific user needs of facilities that cannot afford to install and maintain sophisticated hardware, cloud-based infrastructure has been suggested [44]. This requires reliable and stable Internet connectivity or a hybrid model that stores data locally and synchronises whenever there is stable connectivity [44, 45]. Consequently, the GDN coupled with cloud-based infrastructure is a consideration for Botswana and the developing world, but privacy and confidentiality issues of off-shore data servers must also be considered. Although a viable option in Botswana, literature points to mHealth applications as vulnerable to bandwidth limitations, short battery life-spans, and unreliable access to electricity supply [34, 41]. Reported options to address these challenges include utilisation of broadband Internet, high-speed WiFi and battery packs (power-banks) [34, 40].

The majority (82%) of eRecord systems and the mHealth application support relational database structures (Table 1), open interoperability standards and APIs (Table 2). Similarly, open source eRecord systems have presented opportunities in other settings [34, 35, 40], where MySQL databases and Java applications also enhanced theoretical and technical homogeneity [35]. There were two examples of NoSQL (non-relational databases). NoSQL provide a schema-less database design and offer a more scalable option than traditional relational database management systems (RDBMS) [46, 47]. Despite the lack of a standard query language, limited technical support and lack of maturity [46], large Internet-based companies (Facebook, LinkedIn, Amazon and Google) have adopted NoSQL database systems to address Big Data challenges including optimised information use and scalability [46]. NoSQL databases have been shown to perform better than SQL (relational databases) on the insert and retrieve operations commonly performed on EHR systems [48]. Given the exponential growth of data volumes (structured and unstructured Big Data) across multiple eRecord systems and mHealth applications, NoSQL databases should be the preferred option and are becoming indispensible.

### Data standards, security and unique patient identifier

The lack of agreement on national data and security standards are barriers to interoperability [38–40]. Studies have shown that user preference for free text data over standardised data coding affected semantic interoperability [38, 39]. The majority of eRecord systems in Botswana assign a locally unique identifier to their metadata (Table 3). While this allows data retrieval within each system the absence of nationally unique identifiers remains a barrier to system wide interoperability. Identifiers in the public and private sectors are inconsistent. Added to the problem is synchronisation of patient data collected electronically and in paper systems with different identifiers. This leads to missing patient data, fragmented care and the development of silos of information that cannot be used for patient care in different settings. The need for a unique patient identifier is a goal of the National eHealth Strategy and while biometric identification is a possible solution [49], a simpler and cheaper numeric solution is required.

Non-conformity with available interoperability standards of existing eRecord systems and the mHealth application is another challenge. For example, ISO/IEEE 11073 (Personal Health Device) standards have been implemented in other settings to support linking of mHealth applications to eRecord systems [42]. The need for an accreditation system to ensure proper use of these standards has been noted earlier [44]. Equally important will be alignment of all systems with the DPA to address data security, privacy and confidentiality concerns.

### Capacity building

Consistent with other studies in the developing world, there is a shortage of trained staff to support eRecord systems and existing staff are overburdened [34, 35, 38–40]. The problem is not unique to the developing world, and the American Medical Informatics Association’s 10 by 10 education program, the Informatics Training for Global Health Program of the Fogarty International Center, and the introduction of medical informatics courses at Universities have all tried to address this [50–52]. An important finding of the study was respondents’ lack of understanding of the features and interoperability needs of their eRecord systems. Thus, for six of the 14 questions in the FAIR survey, half of the respondents answered that they were ‘unsure’ (Table 3). This reinforces the need for human resource capacity development already identified in Botswana’s National eHealth Strategy, and underscores the need for interoperability.

### Guidance

Although the National eHealth Strategy was silent on ‘mHealth’, it identified some aspects related to interoperability of eRecord systems. For example, the health-sector interoperability architecture (based on open standards e.g. OpenHIE or OpenHIM) supporting common data registries, terminology services, authentication, and security services, could simplify complexities of interfaces that will be built between mHealth applications and eRecord systems through common APIs (subsection 3.5.3). The Strategy also identified the adoption of global goods (universally available software, services and content) as an appropriate approach (subsection 3.5.3).

Based upon these and other observations, the following guidance for linking mHealth applications and eRecord systems is proffered.

- Given the wide adoption of the HL7 standard (Table 2), an applicable API interoperability standard is the Fast Health Interoperability Resources (FHIR, pronounced “fire”). It offers a contemporary, resource-oriented HTTP interface to search for, create, read, update, and delete FHIR resources using a Representational State Transfer (REST, often referred to as RESTful architectures) based approach to access clinical, administrative, and infrastructure data [53]. Previously reported advantages of RESTful architectures include, “light-weight interfaces that allow for faster transmission and processing of data structures, more suitable for mobile phones and tablet devices. RESTful interfaces also facilitate faster development cycles through their simpler structures” [54].
- Compatibility of mobile device operating systems with eRecord systems and the mHealth application alignment to eRecord systems’ clinical workflows should be addressed within the architecture. Integrating the Healthcare Enterprise (IHE) workflows were reported to promote coordinated use of established standards such as DICOM and HL7 to address specific clinical needs [55].
- The interoperability architecture should further inform acquisition of common health sector infrastructure resources (workstations, mobile devices, connectivity, servers). Cloud-based infrastructure could augment the current GDN services if aligned with Botswana’s eHealth landscape and configured to minimise complexities of connecting data stored locally and in off-site cloud services. Consequently, cloud services could alleviate mobile device limitations of internal storage, processing capacity and short battery lifespan [56]. However, a sustainable funding model is required to support cloud-services. Such arrangements could further support mobile phone gateways for mHealth applications requiring text message (SMS or USSD), voice, or video calls.
- Although a priority, the adoption of global goods should not deter innovation and the development of a home grown EMR as envisioned in the eHealth Strategy. Moreover, accessibility within and outside the GDN should be feasible and user preferences should inform its development.
- Noting the previously reported limitations of relational databases, consideration should also be given to adopting NoSQL databases. This would leverage their: flexibility to store unstructured, semi-structured or structured data; ability to handle large data volumes; ease of use; and support for cloud services often utilised by mobile platforms.
- Security, privacy and confidentiality solutions must be aligned with the DPA. Interoperability standards identified in this study (HL7, ISO/IEEE 11073, DICOM standards) and network security standards prescribed by the DPA are reference points. Consequently, compliance monitoring and accreditation of eRecord systems and mHealth applications to national interoperability standards should be routinely conducted by a standards body in Botswana.

## Limitations

There are few private facilities within Botswana, therefore fewer private facilities were included in the study compared to public facilities. Similarly, the eRecord landscape was not the same across the private and public facilities, so comparing the two was limited. Only one mHealth application (Kgonafalo system) was reported and is not linked to any EMR hence there is no national experience of linking mHealth applications to eRecord systems in Botswana.

## Conclusion

To be sustainable, mHealth applications need to be integrated with diverse eRecord systems within the public and private sectors. The study’s comprehensive situational assessment of the prevailing health ICT landscape provides necessary insight to inform mHealth application and eRecord systems interoperability efforts across the health sector in Botswana. This is an important and recommended step for developing countries in general, where uncertainty of linking mHealth applications to eRecord systems exists.

This study has identified challenges and opportunities for Botswana’s public and private eRecord systems to interoperate between themselves, and with mHealth applications. Proliferation of both eRecord systems and mHealth applications is inevitable and their bi-directional interoperability is essential. Findings from this study are necessary to inform policy makers and health informatics leaders to successfully plan the development of relevant mHealth application and eRecords interoperability frameworks by making better use of available resources and planning for implementation of others.

## Data Availability

The datasets used and/or analysed during the study is available from the corresponding author on reasonable request. The dataset reviewed in this study (the Botswana National eHealth Strategy) is available from the Botswana Health Data Collaborative repository, https://ehealth.ub.bw/bhdc/ehealthstrategy.html.

## Abbreviations

API: Application programming interface
CMS: Central medical stores
CSV: Comma separated values
eHealth: Electronic health
mHealth: Mobile health
DBMS: Database management system
DHIS: District health information system
DICOM: Document imaging and communication in medicine
DPA: Data protection act
eRecord: Electronic record
EMR: Electronic medical record
EHR: Electronic health record
EU: European union
FAIR: Findable, accessible, interoperable, reusable
FHIR: Fast health interoperability resources
GDN: Government data network
GIF: Graphics interchange format
HHR: Health human resource
HICT: Health information communication technology
HIEI: Health information exchange and interoperability
HIMMS: Healthcare information and management systems society
HL7: Health level 7
HTML: Hypertext markup language
ICT: Information communication technology
IHE: Integrating the healthcare enterprise
IT: Information technology
IPMS: Integrated patient management system
ISO/IEEE 11073: International Organization for Standardization/Institute of Electrical and Electronics Engineers 11073
JPEG: Joint photographic experts group
KII: Key informant interviews
MOHW: Ministry of health and wellness
M&E: Monitoring and evaluation
ONC: Office of the national coordinator
OpenMRS: Open medical record system
OpenHIE: Open health information exchange
OpenHIM: Open health information mediator
PDF: Portable document format
PHR: Personal health record
PIMS: Patient integrated management system
PNG: Portable network graphics
RDBMS: Relational database management system
SADACAS: Southern Africa development community accreditation services
SAP: Systems application and products
SMS: Short message service
SOP: Standard operating procedures
SQL: Structured query language
UPI: Unique patient identifier
USSD: Unstructured supplementary service data
WHO: World Health Organisation
XML: Extensible markup language
